# Human umbilical cord mesenchymal stem cell exosomes alleviate acute kidney injury by inhibiting pyroptosis in rats and NRK-52E cells

**DOI:** 10.1080/0886022X.2023.2221138

**Published:** 2023-06-09

**Authors:** Yonghong Wan, Yihang Yu, Chengjun Yu, Jin Luo, Sheng Wen, Lianju Shen, Guanghui Wei, Yi Hua

**Affiliations:** a Department of Urology, Children’s Hospital of Chongqing Medical University, Chongqing, PR China; b Pediatric Research Institute, Children’s Hospital of Chongqing Medical University, Chongqing, PR China; c Chongqing Key Laboratory of Children Urogenital Development and Tissue Engineering, Chongqing Key Laboratory of Pediatrics, Chongqing, PR China; d Ministry of Education Key Laboratory of Child Development and Disorders, Chongqing, PR China; e National Clinical Research Center for Child Health and Disorders, Chongqing, PR China; f China International Science and Technology Cooperation base of Child Development and Critical Disorders, Children’s Hospital of Chongqing Medical University, Chongqing, PR China

**Keywords:** Exosomes, pyroptosis, acute kidney injury, hucMSC

## Abstract

Human umbilical cord mesenchymal stem cells (hucMSCs) have been shown to improve kidney injury. Exosomes have been indicated to be important mediators of renal protection in MSC therapy. In spite of this, the mechanism remains unclear. Our study investigated how exosomes derived from hucMSCs (hucMSC-Ex) improve acute kidney injury (AKI). Exosomes were extracted by using an ultracentrifugation technique and identified by transmission electron microscopy (TEM), nanoparticle tracking analysis (NTA), and Western blot. Twenty-four male SD rats were randomly divided into four groups: sham group, sham + hucMSC-Ex group, ischemia–reperfusion injury (IRI) group, and IRI + hucMSC-Ex group. *In vitro*, we treated rat proximal renal tubular epithelial cell line (NRK-52E) with cisplatin to mimic *in vivo* models of AKI. The NRK-52E cells were treated with or without 160 μg/mL hucMSC-Ex, and 1 µg/mL cisplatin was added after 9 h. Cells were harvested after 24 h. In the IRI group, the levels of serum creatinine (Scr) and blood urea nitrogen (BUN) were increased; renal tubules were dilated, epithelial cells were vacuolated, and collagen fibers were deposited in the renal interstitium. After treatment with cisplatin, the NRK-52E cells displayed pyroptotic morphology characterized by pyroptotic bodies. The protein expression levels of fibronectin, α-smooth muscle actin (α-SMA), vimentin, gasdermin D (GSDMD), caspase-1, interleukin-1 (IL-1β) and NLRP3 in IRI tissues and in cisplatin treatment NRK-52E cells were significantly upregulated. However, after the hucMSC-Ex intervention, kidney injury was effectively improved *in vivo* and *in vitro*. The current study shows that pyroptosis is involved in AKI and that hucMSC-Ex improves AKI by inhibiting pyroptosis.

## 1. Introduction

As a result of acute kidney injury (AKI), renal insufficiency is rapidly developed with high mortality and morbidity rates [1]. In allogeneic kidney transplant patients, ischemia–reperfusion is a common cause of AKI and complications, which is an unavoidable problem during transplantation. Ischemia–reperfusion injury (IRI) may lead to delayed graft function, thereby compromising long-term graft survival and posttransplant recovery [2–4]. Renal fibrosis is a crucial reason for transplanted kidney dysfunction, and IRI is one of the major pathogenic factors associated with renal fibrosis. Strategies for improving fibrotic changes after renal transplantation are the key to successful transplantation. However, effective clinical therapies currently fail to prevent or reverse fibrosis. Therefore, new and active measures are urgently needed.

Stem cells are pluripotent cells that have remarkable abilities to self-renew, regenerate, multiply, and differentiate [5,6]. Additionally, MSCs also have a variety of biological functions, comprising repairing tissue damage, inhibiting inflammatory responses, and modulating the immune system [7,8]. MSCs have achieved good results in treating various diseases, such as renal [9] and liver [10] fibrosis. However, the potential therapeutic mechanism is unclear. Increasing evidence suggests that paracrine effects are the primary mechanism for facilitating renal injury. These therapeutic mechanisms are involved in several biological processes, including angiogenesis and inhibition of apoptosis, inflammation, and fibrosis. Among the secretomes of MSCs, exosomes have been shown to be important mediators of renal protection in MSC therapy.

Living cells secrete exosomes, which are small vesicles with a diameter of about 30–150 nm. They have a lipid bilayer structure and are important information carriers that regulate cell function and gene expression. Substances such as microRNAs and noncoding RNAs are transferred into recipient cells [11]. Compared with MSCs, the advantages of exosomes in treatments include avoiding possible immune rejection, preventing infection, improving safety, and crossing biological barriers. According to the published articles, exosomes derived from human umbilical cord mesenchymal stem cell (hucMSC-Ex) has already been demonstrated to alleviate liver fibrosis [12], skin wound healing [13], and acute myocardial ischemia injury [14], indicating that hucMSC-Ex plays a considerable part in tissue damage and repair.

Pyroptosis is a form of proinflammatory programmed cell death characterized by nuclear shrinkage, the formation and rupture of plasma membrane pores, and inflammatory response [15,16]. It is distinct from apoptosis and necrosis [17]. Pyroptosis is promoted by the NLRP3 inflammasome, a complex assembly. When the NLRP3 inflammasome is activated, caspase-1 is cleaved, and gasdermin D (GSDMD) is cleaved, proinflammatory cytokines are released, and pyroptosis occurs [18]. Activation of caspase-1 is the classical pyroptosis pathway. It induces the inflammatory cell death and secretion of the proinflammatory cytokines interleukin (IL)-18 and IL-1β, thereby promoting removal of pathogens and tissue repair [19]. During AKI, pyroptosis plays a crucial role [20,21]. Hence, we aimed to investigate how exosomes affect pyroptosis during AKI.

## 2. Materials and methods

### 2.1. Isolation and identification of hucMSC-Ex

The collected umbilical cord was sterilized with 75% alcohol (SJZ No.4 Pharmaceutical, Shijiazhuang, China) for 10 s and then rinsed three times with saline. We cut the umbilical cord into small pieces. The chopped umbilical cord tissue was inoculated in 75T (NUNC) culture flasks. They were placed in a 5% CO_2_, saturated humidity, 37 °C incubator. After 3 h, the culture was continued by adding 15 mL of F12 medium (BI) containing 10% fetal bovine serum (ExCell Biology, Shanghai, China). The culture medium was replaced on day 6 of culture. Passage was performed when several blocks of tissue were found to be surrounded by large cell colonies. The above steps were completed by Chongqing Stem Cell Therapy Engineering Technology Research Center (Chongqing, China). P2–P6 hucMSCs were incubated at 37 °C, 5% CO_2_. HucMSCs were routinely cultured in F12 medium (Gibco, Carlsbad, CA) containing 10% fetal bovine serum (Gemini, Calabasas, CA). HucMSC-Ex was extracted from the supernatant of hucMSCs. When 5th generation hucMSCs reached 80% confluence, the culture was changed into the exosome-free culture medium with 10% serum for 48 h. The medium was collected and centrifuged sequentially at 1000 × *g* for 10 min, at 2000 × *g* for 20 min and at 10,000 × *g* for 30 min. After centrifugation, the upper 80% of the supernatant was transferred to Beckman Ultra-Clear Tubes to remove cell debris and residual cells. The hucMSC-Ex were pelleted by ultracentrifugation at 100,000 × *g* for 70 min. For further purification, the supernatant was aspirated layer by layer. The remaining small amount of supernatant was thoroughly mixed with hucMSC-Ex, transferred to Beckman Ultra-Clear Tubes, and ultracentrifuged at 100,000 × *g* for 70 min. The supernatant was decanted, and hucMSC-Ex was suspended in 200 µL of PBS. Large extracellular vesicles in the hucMSC-Ex were removed by filtration through a 0.22 μm pore filter. Determination of HucMSC-Ex protein concentration was carried out using the BCA method according to the directions (MeilunBio, Dalian, China). The morphology of the collected hucMSC-Ex was observed by TEM, and the size distribution of hucMSC-Ex was quantified by NTA. The expression of Alix (1:1000, Proteintech, Rosemont, IL), CD63 (1:1000, Abcam, Cambridge, UK), and TSG101 (1:1000, Proteintech, Rosemont, IL) was detected by western blotting. HucMSC-Ex was stored at −80 °C for subsequent experiments.

### 2.2. Cellular uptake of hucMSC-Ex

First, 400 µg of hucMSC-Ex was resuspended in 400 µL of PBS, and 500 µL of diluent C was added and mixed with hucMSC-Ex. Then, we added 3 µL of PKH26 (Sigma, St. Louis, MO) dye to hucMSC-Ex, mixed well and incubated for 15 min at 37 °C, to label exosomes. The reaction was stopped by adding complete medium. The mixture was placed in a 90Ti tube and ultracentrifuged at 100,000 × *g* for 70 min. HucMSC-Ex was suspended in 400 µL of PBS and stored at −80 °C for later use. NRK-52E cells were cultivated at 37 °C with 5% CO_2_ in DMEM (Gibco, Carlsbad, CA) containing 10% fetal bovine serum (Zeta, Menlo Park, CA). NRK-52E cells were cultured in a 24-well plate, and after the cells adhered, PKH26-labeled exosomes were added, and the cells were fixed after 3, 6, 9, 12 and 24 h. The incubation period at 37 °C was 10 min with the addition of Hoechst. Then, 20 μL of anti-fluorescence quencher were added to the glass slide to mount the slide. HucMSC-Ex uptakes by cells were examined under a confocal microscope.

### 2.3. *In vivo* and *in vitro* experimental models

Male SD rats (190–220 g) were purchased from Chongqing Medical University (Chongqing, PR China). The rats were fed regular food and water and housed in a standard cage at constant temperature and humidity, with a 12-h cycle of light and dark. The rats were randomly divided into four groups (*n* = 6) and treated as follows: (1) in the sham group, sodium pentobarbital (30 mg/kg, ip) was used to anesthetize the rats, and only the bilateral renal pedicles were freed; (2) in the sham + hucMSC-Ex group, the bilateral renal pedicles were dissociated, and 250 µg of hucMSC-Ex was injected into the tail vein immediately afterward; (3) in the IRI group, the renal pedicle was clamped with nondestructive arterial clips for 40 min before reperfusion, and 250 µL of PBS was injected into the tail vein immediately after reperfusion; and (4) in the IRI + hucMSC-Ex group, the renal pedicle was clamped with nondestructive arterial clips for 40 min and then perfused, and 250 µg of hucMSC-Ex was injected into the tail vein immediately after reperfusion. After 24 h, the rats were sacrificed, and kidney tissue and blood were collected for relevant analyses. Bioethical guidelines were followed in all animal procedures, and the Ethics Committee of the Children’s Hospital Affiliated to Chongqing Medical University approved the study (CHCMU-IACUC20220429002).

NRK-52E cells were routinely cultured in DMEM containing 10% fetal bovine serum and 2% penicillin/streptomycin at 37 °C in a cell incubator with 5% CO_2_. NRK-52E cells were cultured in a 10 cm culture dish with or without 160 µg/mL hucMSC-Ex after the cells had adhered. After 9 h, 1 µg/mL cisplatin was added. NRK-52E cells were harvested after 24 h for protein analysis.

### 2.4. Cell viability assay

Cell viability was measured by Cell Counting Kit-8 (MCE, Shanghai, China). NRK-52E cells were cultured at a density of 5000 cells per well in 96-well plates. The cells were treated with different concentrations of cisplatin for 24 h. We added 10 μL of CCK-8 reagent to each well. The cells were incubated for 1 h at 37 °C in an incubator. Microplate readers were used to measure optical density (OD) at 450 nm.

### 2.5. Assessment of renal function

Serum was obtained by centrifugation of rat blood at 7500 × *g* for 10 min at 4 °C. Automatic biochemical analyzers were used to determine the level of serum creatinine (Scr) and blood urea nitrogen (BUN).

### 2.6. Histological analysis

Specimens from each group were fixed in 4% paraformaldehyde, dehydrated, and embedded in paraffin. After sectioning, HE staining, PAS staining, and Masson trichrome staining (Beijing Regen Biotechnology Co., Ltd., Beijing, China) were performed and the stained sections were observed and photographed.

### 2.7. Immunohistochemistry (IHC)

After the paraffin tissue sections were deparaffinized, they were placed in citrate buffer solution, heated 20 min and then cooled to room temperature. After being incubated with endogenous peroxidase blocking solution for 10 min, the sections were washed three times with PBS for 10 min each time, 5% BSA was added for 1 h at room temperature, and the following primary antibodies were added and incubated overnight at 4 °C: smooth muscle actin (1:200, Proteintech, Rosemont, IL), vimentin (1:200, Proteintech, Rosemont, IL), NLRP3 (1:100, Proteintech, Rosemont, IL), caspase 1/P20/P10, (1:200, Proteintech, Rosemont, IL), and IL-1 beta (1:200, Abcam, Cambridge, UK). The sections were washed three times with PBS for 10 min each time, and the reaction-enhancing solution was added. An appropriate amount of peroxidase-labeled goat anti-rabbit IgG was added and incubated at room temperature for 20 min. After being washed three times with PBS for 10 min each time, DAB was added to the sample for several seconds. After observing the color change under the light microscope, the reaction was terminated by placing the sample in deionized water. The nuclei were stained with hematoxylin, and the slides were examined under the light microscope.

### 2.8. Transmission electron microscopy (TEM) and scanning electron microscopy (SEM)

Kidney tissues were fixed with 2.5% glutaraldehyde. NRK-52E cells digested with trypsin were centrifuged at 1000 × *g* for 5 min. The supernatant was discarded and fixed by adding 2.5% glutaraldehyde. The supernatant of treated or NRK-52E cells was discarded and fixed with 2.5% glutaraldehyde. The fixed samples were produced and photographed by Lilai Biotech (Wuhan, China).

### 2.9. Western blot analysis

Kidney tissue and NRK-52E cells were lysed in RIPA lysis buffer (Beyotime, Shanghai, China) containing PMSF and centrifuged at 12,000 × *g* for 20 min at 4 °C. Equal amounts of protein (renal tissue protein: 20 μg, cellular proteins: 15 μg) were separated by SDS-PAGE, the gel was transferred to a PVDF membrane, and blocked with rapid blocking solution (Xin Saimei) for 10 min. The membranes were incubated at 4 °C with primary antibodies against NLRP3 (1:1000, Proteintech, Rosemont, IL), caspase-1 (1:500, Proteintech, Rosemont, IL), GSDMD (1:1000, GeneTex, Irvine, CA), IL-1β (1:500, Abcam, Cambridge, UK), α-smooth muscle actin (α-SMA) (1:1000, Proteintech, Rosemont, IL), vimentin (1:1000, Proteintech, Rosemont, IL), and fibronectin (1:1000, Proteintech, Rosemont, IL) overnight. The membranes were washed three times with TBST for 10 min, diluted secondary antibody (1:10,000, Zenbio, Durham, NC) was added, and the membranes were incubated at room temperature for 1 h. ECL developer (Sorfa, Zhejiang, China) was evenly dropped on PVDF membranes, developed and saved. The gray level of the spots was analyzed with Image Lab software. β-actin was used as an internal reference.

### 2.10. Statistical analysis

All data from different experiments are expressed as mean ± standard deviation (SD). Data were compared among multiple groups using one-way analysis of variance (ANOVA). In all comparisons, *p* < .05 was set for all statistical tests performed on GraphPad 9.0 (GraphPad Software, La Jolla, CA).

## 3. Results

### 3.1. Isolation and identification of hucMSC-Ex

**Figure 1.**
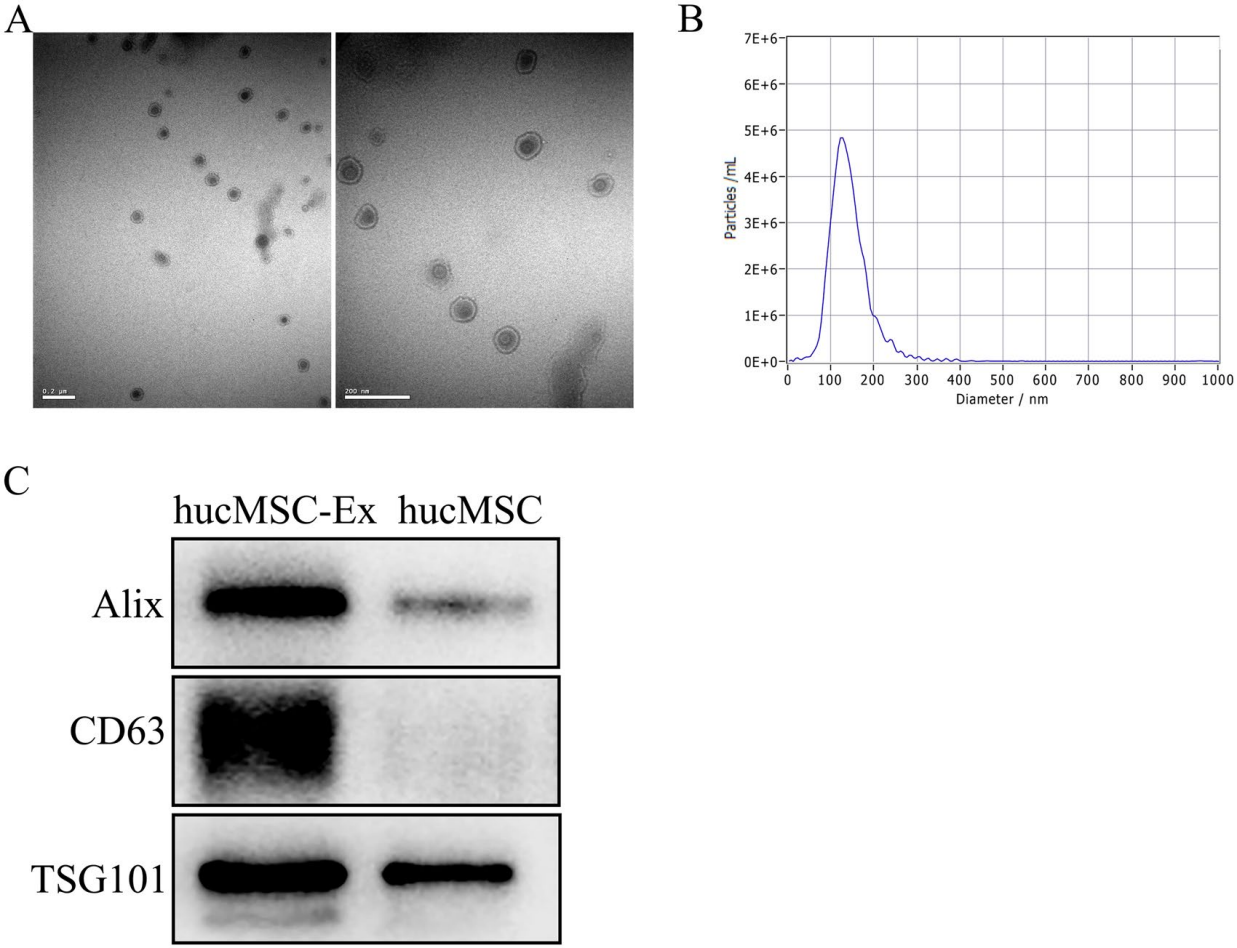
Characterization of hucMSC-Ex. (A) Morphology of hucMSC-Ex under TEM. Scale bar, 200 nm. (B) The mean diameter of hucMSC-Ex was analyzed by NTA. (C) The exosome protein markers Alix, CD63, and TSG101 were detected by Western blot (*n* = 3).

We extracted exosomes from hucMSC supernatants by ultracentrifugation and found that the exosomes were round or oval vesicle-like structures with diameters of approximately 30–150 nm by TEM (Figure 1(A)). NTA indicated that the peak exosome particle size/diameter was 126.7 nm (Figure 1(B)). Western blot analysis showed that hucMSC-Ex expressed the exosomal biomarkers Alix, CD63, and TSG101 (Figure 1(C)). Collectively, these results showed that the isolated material were hucMSC-Ex.

### 3.2. HucMSC-Ex improve AKI induced by IRI in rats

**Figure 2.**
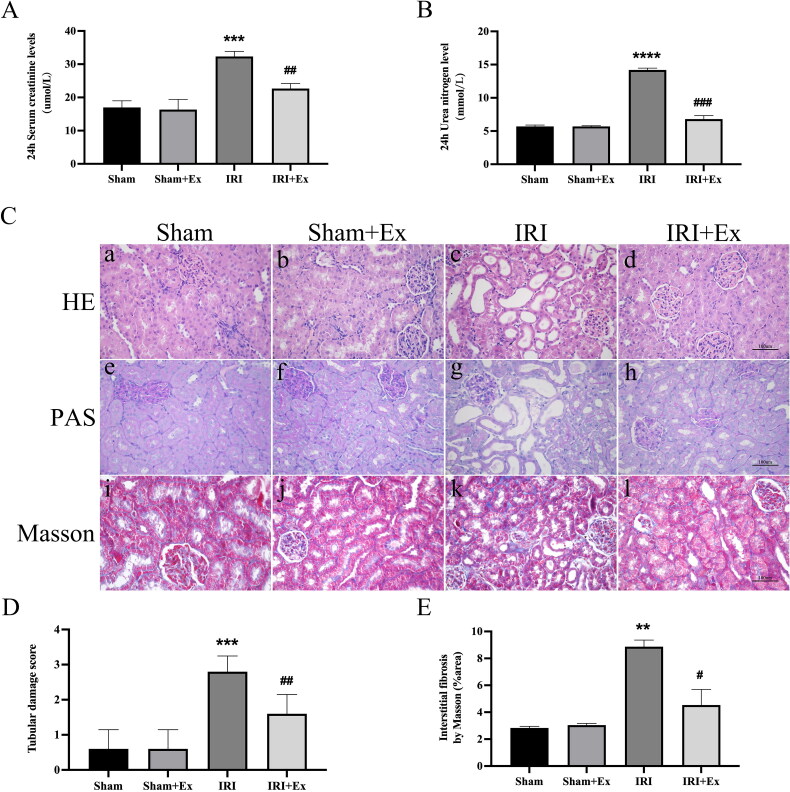
Effects of hucMSC-Ex on kidney histopathology and function in rats with IRI (*n* = 3). (A, B) Scr and BUN values in each group at 24 h. The values are presented as the mean ± SD. ***p < 0.01 vs. sham group; ^##^*p* < 0.1 vs. IRI group; ****p < 0.001 vs. sham group; ^###^*p* < 0.01 vs. IRI group. (C) Histopathological analysis of each group’s kidney. (a–d) Hematoxylin–eosin (HE) staining; (e–h) periodic acid Schiff (PAS) staining; (i–l) Masson’s staining. (D) A tubular damage score was calculated based on the percentage of tubules that displayed cell necrosis, brush loss, cast formation, and tubular dilatation: 0, no damage; 1, <25%; 2, 25–50%; 3, 50–75%; 4, >75%). Magnification: ×400. Quantification of interstitial fibrosis area as percentages of the total area. The values are presented as the mean ± SD. ***p < 0.01 vs. sham group; ^##^*p* < 0.1 vs. IRI group; **p < 0.1 vs. sham group; ^#^*p* < 0.5 vs. IRI group. Sham + Ex: sham + hucMSC-Ex; IRI + Ex: IRI + hucMSC-Ex.

**Figure 3.**
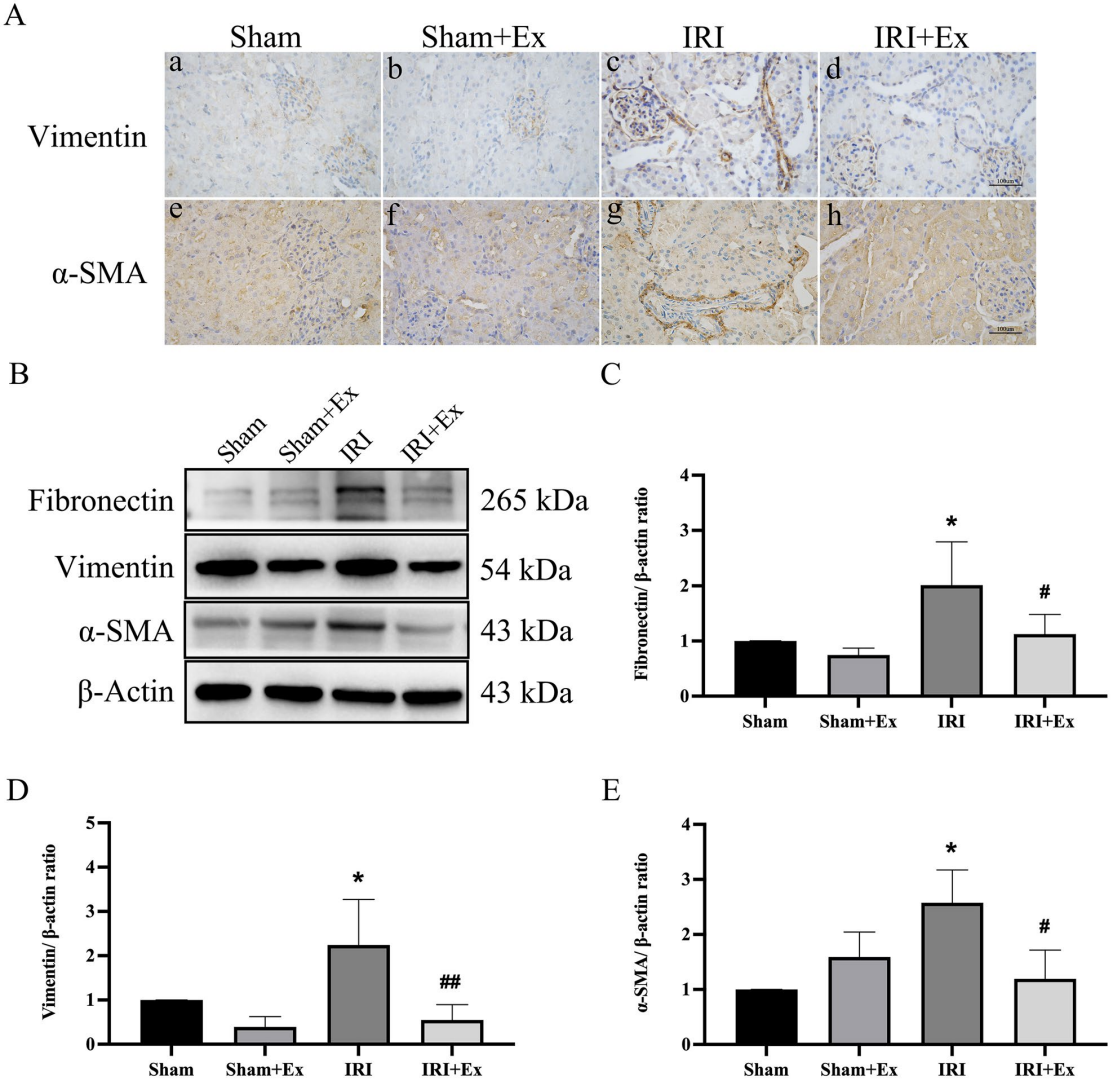
HucMSC-Ex inhibited AKI. (A) Immunohistochemical analysis of each group’s kidney. (a–d) Representative immunohistochemical images of vimentin. (e–h) Representative immunohistochemical images of α-SMA. (B) Examination of fibronectin, vimentin, and α-SMA protein expression. (C–E) An analysis of the relative levels of protein expression in each group (*n* = 3). The values are presented as the mean ± SD. *p < 0.5 vs. sham group; ^#^*p* < 0.5, ^##^*p* < 0.1 vs. IRI group. Sham + Ex: sham + hucMSC-Ex; IRI + Ex: IRI + hucMSC-Ex.

Rats were anesthetized with sodium pentobarbital, and 250 μg of exosomes were injected via the tail vein immediately after IRI. Subsequently, we examined renal function in the four groups. Compared with the sham group, the levels of Scr and BUN were obviously increased in the IRI group and decreased after exosome administration, respectively, indicating that hucMSC-Ex could prevent renal injury in rats (Figure 2(A,B)). HE and PAS staining showed that glomerular and renal tubular cells in the sham group and the sham + hucMSC-Ex group were clear, and there were no noticeable abnormalities in histological morphology. In the IRI group, the proximal and distal convoluted tubules were dilated, the tubular structure was destroyed, exfoliation can be seen in the lumen, the renal tubular epithelial cells were flat, and a large number of inflammatory cells infiltrated in the renal interstitium. The results indicated that hucMSC-Ex could alleviate inflammatory cell infiltration and renal tubular damage in the IRI group. Masson staining showed that in the sham group and sham + hucMSC-Ex group, there was no obvious collagen fiber deposition in the interstitial area, while in the IRI group, the morphological structure of the kidney was destroyed, and collagen fibers were deposited in the interstitial area; when treatment with hucMSC-Ex showed a clear reduction in collagen fiber deposition in the interstitial region (Figure 2(C,D)). Thus, in rats suffering from renal IRI, hucMSC-Ex transplantation can decrease renal tubular injury. IHC showed that the expression levels of vimentin and α-SMA were obviously increased in the IRI group compared with the sham group and the sham + hucMSC-Ex group (Figure 3(A)). Western blot also showed that profibrotic proteins (including fibronectin, vimentin, and α-SMA) expression levels were increased in the IRI group but were decreased after hucMSC-Ex treatment (Figure 3(B–E)). In conclusion, hucMSC-Ex can improve the progression of AKI induced by IRI.

### 3.3. HucMSC-Ex improve AKI by inhibiting pyroptosis

**Figure 4.**
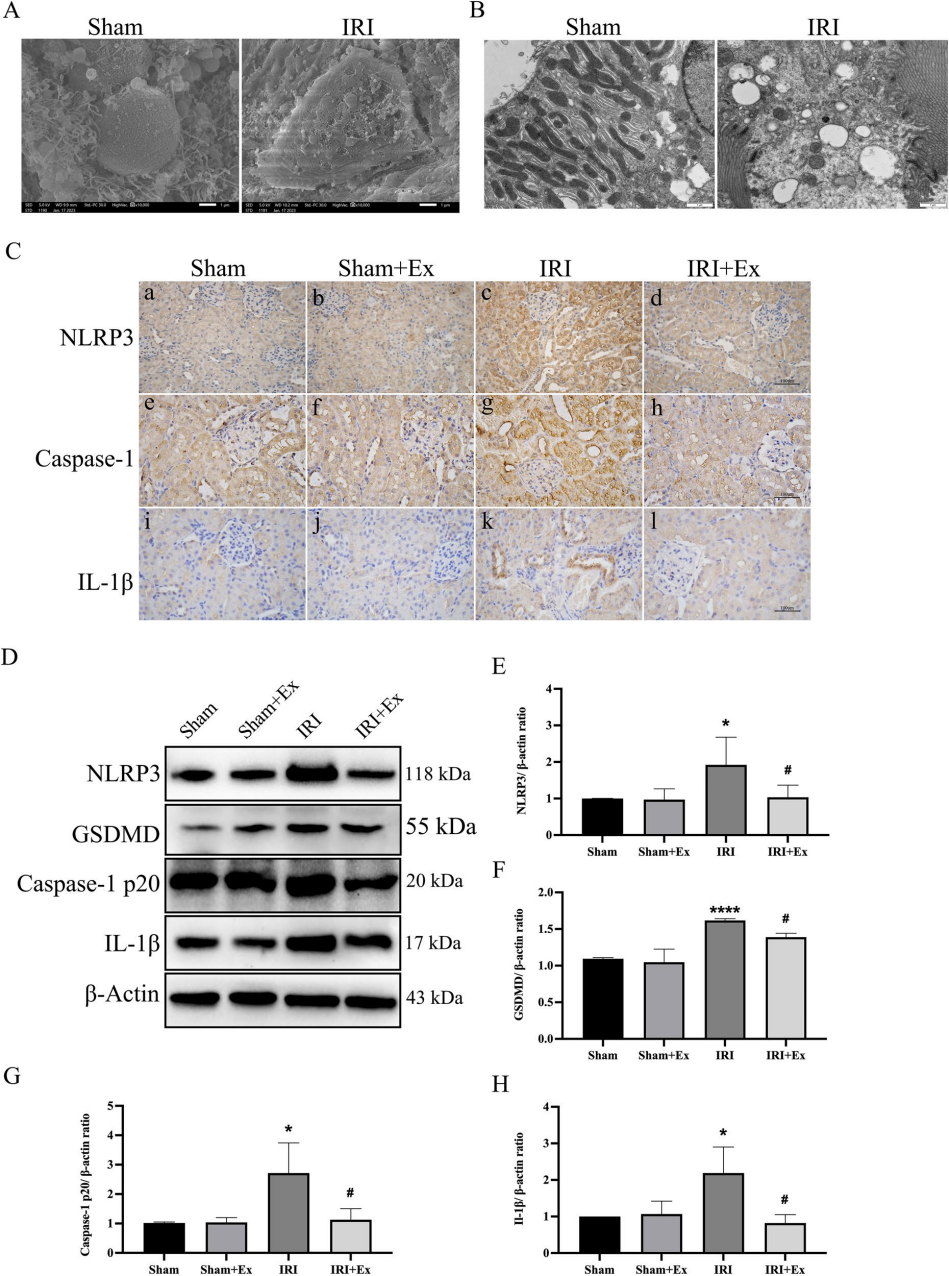
HucMSC-Ex inhibits renal pyroptosis *in vivo*. (A) Representative scanning electron micrographs of pyroptosis in kidney. (B) Representative transmission electron microscopes of pyroptosis in kidney. (C) Immunohistochemical analysis of each group’s kidney. (a–d) Immunohistochemical analysis of NLRP3. (e–h) Immunohistochemical analysis of caspase-1. (i–l) Immunohistochemical analysis of IL-1β. (D–H) Western blot analysis of NLRP3, GSDMD, caspase-1 p20, and IL-1β expression in each group (*n* = 3). The values are presented as the mean ± SD. *p < 0.5, ****p < 0.001 vs. sham group; ^#^*p* < 0.5 vs. IRI group. Sham + Ex: sham + hucMSC-Ex; IRI + Ex: IRI + hucMSC-Ex.

To investigate the reason for hucMSC-Ex improving AKI and considering the important role of pyroptosis in AKI, we examined pyroptosis levels in the four groups. First, we used SEM, and observed that the sham group had no significant damage, while the IRI group had obvious damage, disordered cell arrangement, and visible pyroptotic bodies (Figure 4(A)). Subsequently, we observed mitochondrial swelling and cell membrane disruption in the IRI group by using TEM (Figure 4(B)). Considering that excessive inflammation leads to pyroptosis, and also to clarify the relationship between AKI and pyroptosis, we examined the activity of the pyroptosis pathway. IHC (Figure 4(C)) showed that the expression levels of NLRP3, caspase-1 p20, and IL-1β in the kidney tissues of the IRI group were increased compared with those in the sham group, the sham + hucMSC-Ex group and IRI + hucMSC-Ex, suggesting that hucMSC-Ex can inhibit pyroptosis and improve AKI induced by IRI. To further prove our results, we also carried out western blot to evaluate the expression of NLRP3, caspase-1, GSDMD, and IL-1β (Figure 4(D–H)), and the results were consistent with the IHC.

### 3.4. Cellular uptake of hucMSC-Ex

**Figure 5.**
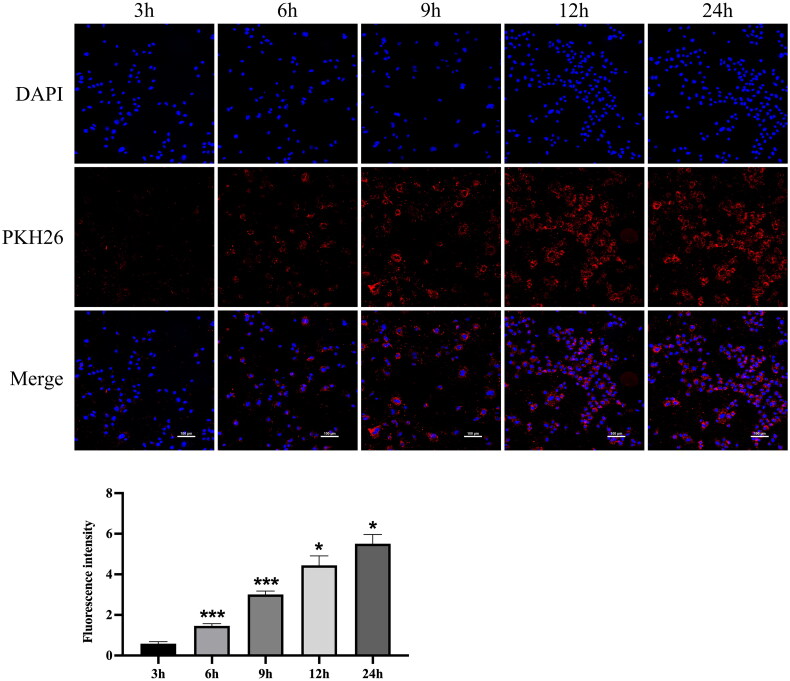
Cellular uptake of hucMSC-Ex (*n* = 3). Representative confocal images of the cellular uptake of pkh26-labeled hucMSC-Ex and fluorescence intensity analysis (*n* = 3). The values are presented as the mean ± SD. Compared with the previous time point, *p < 0.5, ***p < 0.01 PKH26-labeled hucMSC-Ex: red; DAPI-labeled nucleus: blue.

Cellular uptake is a prerequisite for drug delivery. Since tubular epithelial cells are the main cell type affected during AKI, we used NRK-52E cells to perform subsequent experiments. Therefore, we examined the uptake of hucMSC-Ex by NRK-52E cells. Confocal analysis showed that hucMSC-Ex was easily taken up by NRK-52E cells. The fluorescence intensity of hucMSC-Ex in cells increased with time. At 9 h, most cells had successfully taken up exosomes, and at 6 h, some cells had taken up exosomes (Figure 5). Therefore, in all subsequent experiments, we added exosomes first, followed by cisplatin 9 h later.

### 3.5. HucMSC-Ex improve cisplatin-induced injury in NRK-52E cells

**Figure 6.**
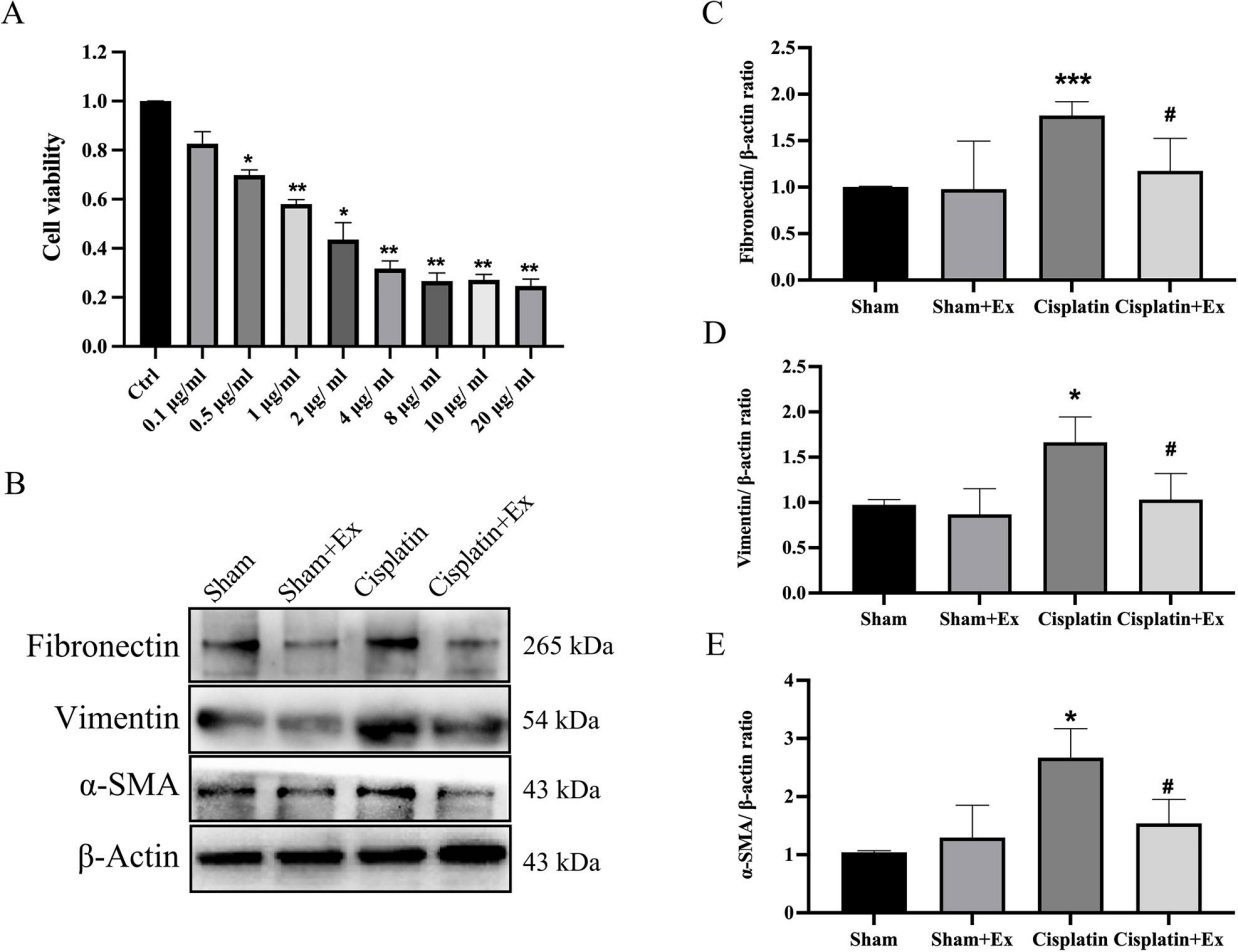
HucMSC-Ex inhibit renal fibrosis *in vitro*. (A) Eight different concentrations of cisplatin were used to treat NRK-52E cells for 24 h, and CCK-8 was used to assess cell viability. (B–E) Western blot analysis of fibronectin, vimentin, and α-SMA expression (*n* = 3). The values are presented as the mean ± SD. *p < 0.5, ***p < 0.01 vs. sham group; ^#^*p* < 0.5 vs. IRI group. Sham + Ex: Sham + hucMSC-Ex; IRI + Ex: IRI + hucMSC-Ex.

According to studies, cisplatin can induce NRK cell pro-fibrotic changes. Therefore, we treated NRK-52E cells with cisplatin to mimic *in vivo* models of AKI and further explore the molecular mechanism of cisplatin toxicity. The viability of NRK-52E cells treated with 1 µg/mL cisplatin decreased to 58% when compared to that of the control group (Figure 6(A)). Therefore, in the following experiments, we treated NRK-52E cells with 1 µg/mL cisplatin to test whether cisplatin reduces cell viability by causing NRK-52E cells fibrotic changes. We found that the expression levels of fibrosis-related proteins containing fibronectin, vimentin, and α-SMA were increased in the cisplatin group, while their expression levels were decreased by treatment with hucMSC-Ex (Figure 6(B–E)), indicating that HucMSC-Ex can improve cisplatin-induced injury in NRK-52E cells.

### 3.6. HucMSC-Ex inhibits pyroptosis and improves NRK-52E cells injury

**Figure 7.**
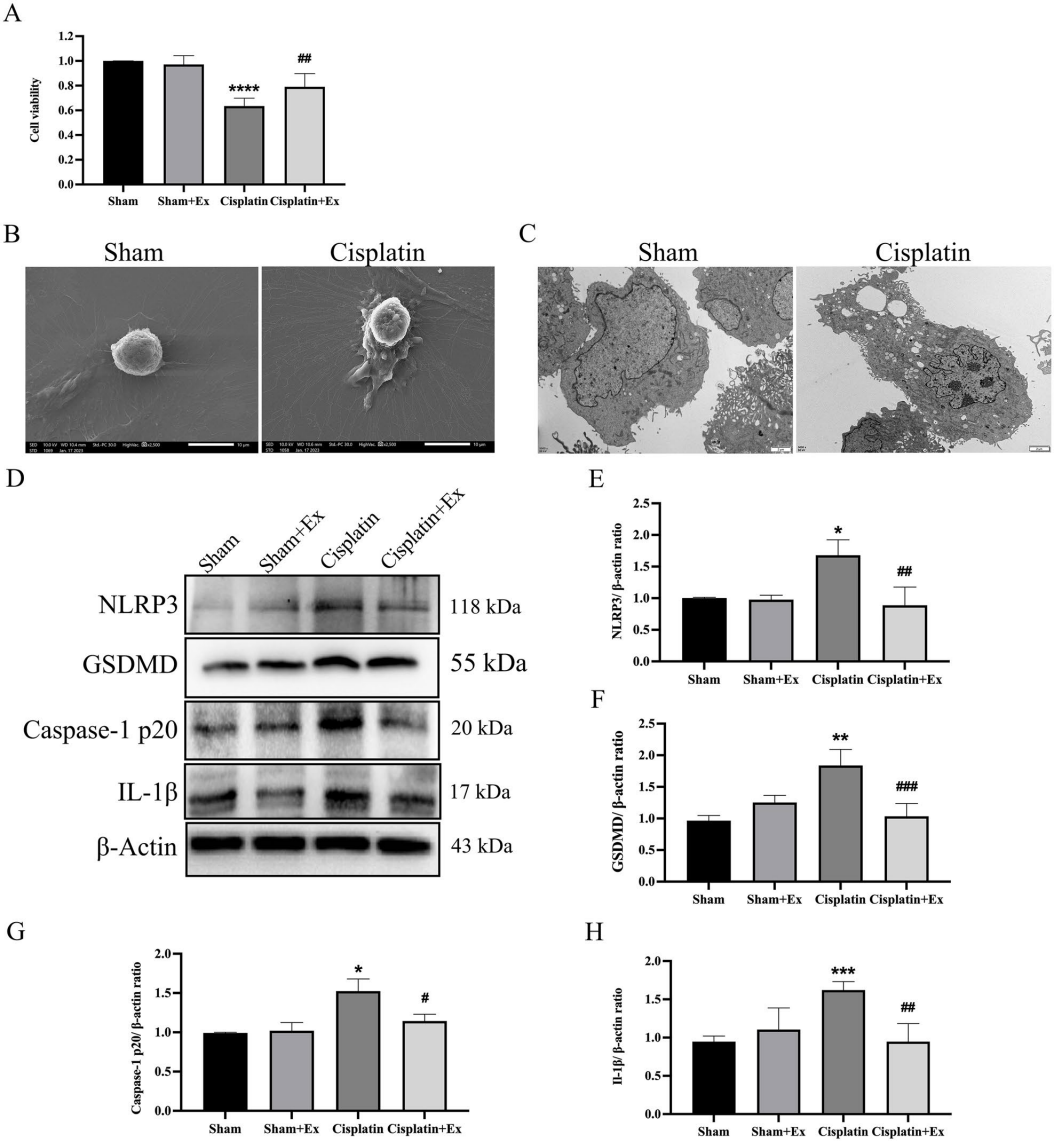
HucMSC-Ex inhibit renal pyroptosis *in vitro*. (A) NRK-52E cells were treated with 1 µg/mL cisplatin and 160 µg/mL hucMSC-Ex, and cell viability was detected by using CCK-8. (B) Representative scanning electron micrographs of pyroptosis in NRK-52E cells. (C) Representative transmission electron microscopes of pyroptosis in NRK-52E cells. (D–H) Western blot analysis of NLRP3, GSDMD, caspase-1 p20, and IL-1β expression (*n* = 3). The values are presented as the mean ± SD. *p < 0.5, **p < 0.1, ***p < 0.01 vs. sham group; ^#^*p* < 0.5, ^##^*p* < 0.1, ^###^*p* < 0.01 vs. IRI group. Sham + Ex: Sham + hucMSC-Ex, IRI + Ex: IRI + hucMSC-Ex.

Moreover, we also discovered that hucMSC-Ex could attenuate the toxicity of cisplatin and increase the survival rate of cells by CCK-8 (Figure 7(A)). After cisplatin treatment, NRK-52E cells displayed a pyroptotic morphology characterized by pyroptotic bodies (Figure 7(B)), and massive pore formation of membranes can be observed (Figure 7(C)). To further explore the molecular mechanism, we detected the expression levels of pyrolysis-associated proteins, including NLRP3, caspase-1 p20, GSDMD, and IL-1β. Compared with the sham group, the cisplatin group showed increased expression of NLRP3, caspase-1 p20, GSDMD, and IL-1β, and their expression levels were decreased after treatment with hucMSC-Ex (Figure 7(D–H)), describing that hucMSC-Ex can inhibit pyroptosis and improve NRK-52E cells injury.

## 4. Discussion

Organ transplantation is one of the most dramatic and important achievements in medicine in the twentieth century and the most effective treatment for end-stage diseases [22]. However, due to the lack of donor organs, a large number of patients must choose marginal donors or donors who donate after circulatory death. In such transplant patients, the protection of allograft function is particularly crucial, especially in renal fibrosis after IRI. MSCs can respond to specific triggers with differentiation to mature cells with consequent tissue regeneration [23]. MSCs could represent a revolutionary treatment for patients with fibrotic evolution. Recent studies have shown that hucMSCs have the potential to repair kidney damage, which is mainly related to their secretion [24]. Among the secretomes of MSCs, exosomes have been shown to be critical mediators of renal protection in MSC therapy. Therefore, we selected hucMSC-Ex to treat the IRI rat model and examined the related mechanism. Our results suggest hucMSC-Ex expresses exosomal surface markers, such as Alix, CD63, and TSG101. TEM showed that the cell bodies were round or elliptical with diameters of approximately 50–150 nm. Nanoparticle tracking analysis (NTA) indicated that the peak exosome particle size/diameter was 126.7 nm. The *in vitro* uptake experiments of PKH26-labeled exosomes demonstrated that hucMSC-Ex could be effectively taken up by cells.

*In vivo*, we used IRI to construct an animal model of AKI. Compared with the traditional UUO model, this model can better simulate AKI caused by IRI after kidney transplantation and is similar to the clinical case study. *In vitro*, we treated NRK-52E cells with cisplatin to mimic an *in vivo* model of AKI. Compared with other fibrosis models, this experimental model is more stable. Comparing Scr and BUN at 24 h after surgery, we found that the level of Scr and BUN in the IRI group was increased and decreased after exosome intervention. Moreover, vascular collapse, renal tubular dilation or atrophy, inflammatory cell infiltration, extracellular matrix deposition, and collagen fiber deposition were observed in the interstitial region in rats in the IRI group. HucMSC-Ex can alleviate the renal tubular injury, indicating that hucMSC-Ex has a protective effect against renal injury.

Scarring is characterized by fibrosis and results from an increase in activated myofibroblasts that deposit excess extracellular matrix [25]. Activated myofibroblasts normally express secrete collagen and desmin, which can be identified by examining the expression of α-SMA and vimentin. During fibrosis, fibroblasts change their characteristics, express different extracellular matrix proteins, and synthesize more collagen and proteoglycans (tenascin, laminin, and fibronectin) [26]. Therefore, we chose α-SMA, vimentin and fibronectin to validate the model. IHC and Western blot indicated that the expression levels of fibrosis-related proteins were increased in the IRI group but decreased after hucMSC-Ex treatment. Thus, we believed that hucMSC-Ex had a protective effect against IRI-induced renal fibrotic changes.

Pyroptosis is characterized by the rupture of the plasma membrane and the release of intracellular proinflammatory substances [27]. NLRP3, ASC, and pro-caspase-1 constitute the NLRP3 cytoplasmic multi-protein molecular platform [28]. Inflammation or injury triggers NLRP3 activation, which leads to caspase-1 activation, which then induces IL-18 and IL-1β maturation. At the same time, active caspase-1 can cleave GSDMD, which is a key molecule that induces pyroptosis [29]. Pyroptosis can lead to cell swelling, cell rupture and the formation of pores in the plasma membrane [30]. Therefore, we used SEM and TEM to observe renal tissue in rats and NRK-52E cells. We observed pyroptosis in kidneys with IRI and in NRK-52E cells treated with cisplatin. During pyroptosis, the outcome of cell lysis is mediated by caspase-1 [31–40]. The secretion of inflammatory cytokine IL-1β depends on the activation of caspase-1. IL-1β is a powerful endogenous pyrogen that stimulates fever, the expression of various cytokines and chemokines, and leukocyte tissue migration [41]. Pyroptosis is activated by the NLRP3/caspase-1 axis and has been associated with a large number of kidney injuries and diseases [42,43]. Therefore, we speculated that the pyroptosis-induced NLRP3/caspase-1 axis might be involved in AKI. Moreover, HucMSC-Ex inhibited pyroptosis and improved AKI. Our research demonstrated that after hucMSC-Ex treatment, the pyroptosis-related proteins GSDMD, caspase-1, NLRP3, and IL-1β expression levels were decreased in renal tissue of IRI rats, suggesting that hucMSC-Ex inhibits pyroptosis. Cisplatin has been shown to cause renal injury, and we treated NRK-52E cells with cisplatin for 24 h to construct a AKI cell model. Western blot analysis indicated that the fibrosis-related proteins fibronectin, vimentin, and α-SMA expression levels were increased in the cisplatin group but were decreased after hucMSC-Ex treatment. The expression levels of the pyroptosis-related proteins NLRP3, GSDMD, caspase-1, and IL-1β in the renal tissue of IRI rats were decreased after treatment with hucMSC-Ex. In the *in vitro* experiment, we observed the same outcomes. Therefore, we believe that hucMSC-Ex has a protective effect on AKI by improving pyroptosis.

## 5. Conclusions

The current study shows that pyroptosis is involved in AKI and that hucMSC-Ex improves AKI by inhibiting pyroptosis. Continued attempts to elucidate the target molecules are activated by pyroptosis. It helps to clarify further the cellular and molecular mechanisms of the protective effect of hucMSC-Ex on acute kidney disease.

## Data Availability

The datasets used and/or analyzed during the current study are available from the corresponding author on reasonable request.
